# Serum Levels of Alpha7 Nicotinic Acetylcholine Receptor and Their Association With Cardiovascular Autonomic Function Tests in Healthy Young Adults

**DOI:** 10.7759/cureus.94794

**Published:** 2025-10-17

**Authors:** Shanmugavel Karthik, Mohan Elakiya, Kandan Balamurugesan

**Affiliations:** 1 Department of Physiology, Jawaharlal Institute of Postgraduate Medical Education and Research (JIPMER), Puducherry, IND; 2 Department of Medicine, Jawaharlal Institute of Postgraduate Medical Education and Research (JIPMER), Puducherry, IND

**Keywords:** alpha7 nicotinic acetylcholine receptor, deep breathing, heart rate variability, isometric handgrip, sex characteristics

## Abstract

Introduction: The autonomic nervous system controls various physiological functions through sympathetic and parasympathetic pathways, mediated by adrenergic and cholinergic receptors. Among these, the alpha7 nicotinic acetylcholine receptor (alpha7 nAChR) plays a key modulatory role in neuronal and nonneuronal systems. This study aimed to investigate the relationship between serum alpha7 nAChR levels and cardiovascular autonomic function in healthy young adults with a normal body mass index, focusing on gender differences.

Methods: Thirty-three participants (both male and female participants) underwent cardiovascular autonomic assessments, including resting heart rate variability (HRV), the expiration-to-inspiration (E:I) ratio from deep breathing, and diastolic blood pressure response during isometric handgrip. Serum alpha7 nAChR levels were measured using Enzyme-Linked Immunosorbent Assay.

Results: Although male participants showed higher mean alpha7 nAChR levels than female participants, this difference was not statistically significant. No significant correlation was observed between alpha7 nAChR levels and resting HRV parameters. However, alpha7 nAChR levels showed a significant positive correlation with the E:I ratio during deep breathing in male participants.

Conclusions: Although overall alpha7 nAChR levels showed no significant difference between male and female participants, their association with parasympathetic reactivity in male participants suggests a possible sex difference in autonomic regulation that merits further exploration.

## Introduction

The autonomic nervous system (ANS) regulates many vital physiological processes through its sympathetic and parasympathetic branches. The vagus nerve, a key component of the parasympathetic system, innervates most internal organs and helps maintain homeostasis by acting through muscarinic and nicotinic receptors [1]. Among these, the alpha7 nicotinic acetylcholine receptor (alpha7 nAChR) stands out for its unique monomeric structure [2] and important roles in cognition, mood, and the cholinergic anti-inflammatory response [2,3].

Alpha7 nAChR is a membrane protein found not only in neurons but also in nonneuronal cells such as astrocytes, microglia, immune cells, thymus, spleen, and adrenal medulla [4]. It plays a critical role in the cholinergic anti-inflammatory reflex [3] and has been linked to cardiac function through its presence in autonomic ganglia [4], as well as to endothelial function [5], cardiac remodeling [6], and inflammation modulation. Notably, alpha7 nAChR can upregulate matrix metalloproteinase-2 (MMP2) via the alpha7 nAChR-JNK pathway in macrophages and vascular smooth muscle cells [7]. MMP2 is a vital proteolytic enzyme involved in shedding ectodomain components of macrophage membrane proteins [8]. This suggests a plausible mechanism by which fragments of alpha7 nAChR may be released into the circulation and become measurable in serum despite their membrane-bound nature.

While alpha7 nAChR is widely recognized for its roles in neuronal signaling and immune modulation, it is also expressed in autonomic ganglia [4], key structures of the peripheral ANS. However, its specific functional role in autonomic regulation, particularly in humans, remains poorly characterized.

While some membrane protein biomarkers, such as Toll-like receptor 8 [9], vascular cell adhesion molecule-1 [10], and tumor necrosis factor receptor [11], are widely used to reflect immune or vascular inflammation, they do not capture cholinergic modulation or autonomic function. In contrast, alpha7 nAChR represents a unique intersection of neural, immune, and autonomic pathways via the cholinergic anti-inflammatory reflex and may provide more specific information in this regard.

Moreover, to understand its relevance in disease, it is important to first understand its levels in a healthy state and how common factors like age, sex, and body mass index (BMI) might influence it. To the best of our knowledge, there is a scarcity of human studies examining how circulating alpha7 nAChR relates to measures of cardiovascular autonomic function such as heart rate variability (HRV). This study addresses this gap by exploring these associations.

Given the established influence of gender and BMI on both autonomic regulation and systemic inflammatory responses, these variables were included in the current study to explore their potential associations with serum alpha7 nAChR levels. Although direct evidence of their effect on serum alpha7 nAChR is limited, their known physiological roles in autonomic regulation and systemic inflammation support their inclusion in this exploratory work.

If validated, serum alpha7 nAChR could emerge as a useful biomarker to identify individuals with altered autonomic or immune regulation, potentially aiding risk assessment or monitoring in conditions such as cardiovascular disease, metabolic syndrome, and chronic inflammatory disorders. As a first step, the present exploratory study aims to find the levels of the alpha7 nAChR in healthy participants and their relation with autonomic function tests.

## Materials and methods

Participant population

This observational analysis utilized data from a larger interventional study investigating the effects of transcutaneous auricular vagus nerve stimulation on autonomic function and quantitative EEG markers in healthy volunteers. The study protocol received approval from the Institutional Ethics Committee (no: JIP/IEC/2022/031) at the Jawaharlal Institute of Postgraduate Medical Education and Research (JIPMER), Puducherry, and was conducted in accordance with the Declaration of Helsinki.

A total of 33 participants were included, slightly exceeding the commonly accepted minimum of 30 for pilot or exploratory studies. The sample size was originally calculated for the primary outcomes of the parent study; convenience sampling was used to enroll volunteers who met the inclusion criteria, without randomization. Recruitment occurred through community outreach and among healthcare workers. Eligible healthy adult volunteers aged 18-35 years, with a BMI between 18.0 and 22.9 kg/m², who self-reported, were enrolled after clinical screening at the Department of Medicine, JIPMER. We selected this group of young, healthy individuals with normal BMI to establish baseline alpha7 nAChR levels in a physiologically stable population. This approach also helps minimize the confounding effects of aging and obesity, both of which are known to significantly impact autonomic function. To minimize hormonal variability as a confounding factor, female participants were assessed during the early proliferative phase of their menstrual cycle. Exclusion criteria included any history of cardiovascular disease, neurological disorders, diabetes mellitus, hypertension, asthma or chronic pulmonary disease, active peptic ulcer disease, chronic medication use, contraceptive use, regular physical exercise, or yoga practice, as these factors can influence autonomic function test parameters. Physical activity levels were not recorded, and this limitation is addressed in the discussion.

Study procedure

Written informed consent was obtained from all participants before enrollment. The study was conducted in the Neurophysiology Laboratory of the Department of Physiology at JIPMER. Participants were instructed to abstain from nicotine, alcohol, and caffeinated beverages for 24 hours before testing to reduce potential acute effects. All recordings were performed between 9:00 and 11:00 A.M. to minimize diurnal variation. BMI was calculated using the Quetelet Index at the time of assessment by dividing body weight in kilograms (measured with a digital weighing machine with 0.1 kg sensitivity) by height in square meters (measured using a wall-mounted stadiometer with 0.1 cm sensitivity). Mental well-being was assessed using the WHO-5 Well-Being Index, a validated tool developed by the World Health Organization [12].

This study is embedded within a broader protocol involving vagus nerve stimulation and EEG recordings. To minimize participant burden and ensure consistency across protocols, we selected three core autonomic tests, resting HRV, isometric handgrip, and deep breathing, representing overall sympathovagal balance, sympathetic reactivity, and parasympathetic reactivity, respectively. Participants rested supine for 15 minutes before 15-minute Lead II ECG recordings were obtained using a Galileo-NT EB Light physiograph (Florence, Italy). The ECG setup employed a sensitivity of 100 mm/µV, with a 70 Hz low-pass filter, a 0.3 Hz high-pass filter, a 512 Hz sampling rate, and a 50 Hz notch filter to minimize noise. HRV parameters and autonomic reactivity measures, expiration-to-inspiration (E:I) ratio during paced deep breathing, and the change in diastolic blood pressure (ΔDBP) during the IHG test were analyzed.

Serum alpha7 nAChR levels were measured using a sandwich Enzyme-Linked Immunosorbent Assay kit (ELK Biotechnology, Denver, Colorado), following the manufacturer's instructions. Preliminary dilution experiments determined that a 1:10 dilution provided results consistently within the assay's validated detection range (0.32-20 ng/mL). The assay sensitivity was 0.122 ng/mL, and quantification utilized a four-parameter logistic standard curve with excellent fit (R² = 1.000).

Data analysis

ECG signals were manually inspected by trained researchers to remove artifacts and ectopic beats. In addition, automated R-wave peak detection and artifact identification were performed using the open-source Brainstorm software (version 2.0, Computational Neuroscience Laboratory, University of Southern California, Los Angeles, California), which was used to simultaneously analyze EEG and ECG data. Although a formal interrater reliability assessment was not conducted, all data cleaning procedures followed standardized protocols implemented by the same research team. Clean 300-second ECG segments were analyzed for RR intervals using LabChart Pro software (AD Instruments, Bella Vista, Australia). Time-domain HRV metrics (standard deviation of NN intervals (SDNN), root mean square of successive differences, and NN50), frequency-domain parameters (low-frequency (LF) power, high-frequency (HF) power, and LF/HF ratio), and nonlinear measures (Sample Entropy, Approximate Entropy, and Poincaré plot parameters SD1 and SD2) were calculated with the Kubios HRV software (version 3.5, University of Eastern Finland) [13].

Parasympathetic reactivity was assessed through six cycles of paced breathing (five seconds inhale, five seconds exhale). The E:I ratio was calculated by dividing the mean of the longest RR intervals during exhalation by the mean of the shortest RR intervals during inhalation. Sympathetic reactivity was evaluated using the IHG test, where participants maintained 30% of their maximum voluntary contraction for four minutes. Blood pressure (BP) was measured at baseline, then every minute during handgrip, and immediately before release. ΔDBP was defined as the difference between the maximum diastolic pressure during handgrip and baseline.

Statistical analysis

Demographic variables, including age, BMI, and serum alpha7 nAChR levels, were summarized as means ± standard deviations. The Shapiro-Wilk test assessed the normality of continuous variables. Gender distribution was assessed using the chi-square test (χ²). Between-group comparisons by sex used Welch's t-test for normally distributed data and Mann-Whitney U tests for nonnormal variables, with effect sizes reported as Hedges' g or rank-biserial correlation. All data points were screened for outliers using boxplots. A small number of outliers were detected but were retained for analysis after confirming their biological plausibility and minimal impact on overall results through sensitivity analyses. Missing data were not present in the final dataset.

To improve the robustness of correlation estimates, we applied 1,000-sample bias-corrected and accelerated bootstrap resampling to generate 95% confidence intervals (CIs) for key associations. This nonparametric approach accounts for potential nonnormality and small sample limitations. HRV and autonomic parameters, showing nonnormal distributions, were reported as medians with CI calculated using the Hodges-Lehmann estimator. Given the exploratory nature and sample size, no multiple comparisons correction was applied, but results are interpreted cautiously with emphasis on effect sizes and CIs.

Post hoc analysis of covariance (ANCOVA) analyses were performed to examine sex differences in BP while controlling for BMI. A linear regression analysis including an Age × Gender interaction term was performed to assess whether the association between age and alpha7 nAChR levels differed by gender. Associations between serum alpha7 nAChR levels and autonomic parameters were examined using Spearman's partial correlation, adjusting for age and BMI to control for confounding. Other potential covariates, such as diet and lifestyle factors, were not included due to a lack of available data; this limitation is acknowledged in the Discussion section. We conducted sensitivity analyses by rerunning the correlation analyses after excluding potential outliers identified using visual inspection and statistical thresholds (values beyond 1.5× interquartile range). The direction and magnitude of the associations remained consistent. This suggests that the observed trends are robust and not unduly influenced by extreme values. All statistical tests were two-tailed, with significance set at p < 0.05. Analyses were performed using R software (version 4.0.3; R Foundation for Statistical Computing, Vienna, Austria) and RStudio (version 1.3.1073; Posit Software, Boston, MA).

## Results

The study included 33 healthy young adults (18 men and 15 women; Table 1). The mean age of participants was 22.76 ± 3.38 years, with male participants averaging 21.89 ± 1.78 years and female participants 23.80 ± 4.49 years. The average BMI was 22.42 ± 2.47 kg/m² (men: 21.76 ± 2.67, women: 23.21 ± 2.01). There were no statistically significant gender differences in age (t = 1.55, p = 0.139, Hedges' g = 0.57) or BMI (t = 1.78, p = 0.085, g = 0.60). The mean WHO-5 Well-Being Index score was 67.39 ± 22.36, with male participants scoring 72.00 ± 21.30 and female participants 61.87 ± 23.07, showing no significant gender difference (t = -1.30, p = 0.204, g = -0.45). Gender distribution did not differ significantly from equal proportions (χ² = 0.273, p = 0.602, φ = 0.09). Effect sizes (Hedges' g) ranged from small to moderate, indicating modest, nonsignificant sex-related differences. Overall, the sample was well-balanced and representative of healthy young adults. Given the narrow age range of participants (18-35 years), subgroup analyses by age were not conducted; however, age was included as a covariate in correlation analyses to account for potential residual effects. Outlier screening identified a few data points exceeding ±3 standard deviations, which were retained due to their plausibility and minimal impact on the findings.

**Table 1:** Demographic characteristics of healthy young participants Values are presented as mean ± standard deviation. Gender distribution was assessed using the chi-square test (χ²). Between-group comparisons (male vs. female) were performed using Welch’s t-test. Effect size for frequency is reported as phi (φ) from Cramér’s V. Effect size for continuous variables is reported as Hedges’ g. A p value of <0.05 was considered statistically significant. WHO-5 score has been referred from [12] BMI: body mass index; WHO-5 score: World Health Organization Well-Being Index (five items) score

Parameter	Participants (n = 33)	Male (n = 18)	Female (n = 15)	Test statistic	df	p value	Effect size
Frequency (n)	33 (100%)	18 (54.5%)	15 (45.5%)	χ² = 0.273	1	0.602	φ = 0.09
Age (years)	22.76 ± 3.38	21.89 ± 1.78	23.8 ± 4.49	t = 1.55	18	0.139	g = 0.57
BMI (kg/m^2^)	22.42 ± 2.47	21.76 ± 2.67	23.21 ± 2.01	t = 1.78	31	0.085	g = 0.60
WHO-5 score	67.39 ± 22.36	72.00 ± 21.3	61.87 ± 23.07	t = -1.30	29	0.204	g = -0.45

Serum alpha7 nAChR levels averaged 137.04 ng/mL (95% CI: 128.78-145.31) across all participants (Table 2). The mean levels were 138.24 ng/mL (126.81-149.67) in men and 135.61 ng/mL (122.08-149.13) in women. The between-group difference was not statistically significant (mean difference = -2.63 ng/mL; 95% CI: -19.63 to 14.36; Welch’s t-test: t = -0.32, df = 29, p = 0.754), with a small effect size (Hedges’ g = -0.11; Table 2). Serum alpha7 nAChR levels exhibited moderate variability, with coefficients of variation of 16.6% in men, 18.0% in women, and 17.0% in the combined sample. These results indicate consistent measurements across participants and suggest minimal gender-based biological differences in serum alpha7 nAChR levels among healthy young adults.

**Table 2:** Serum levels of alpha7 nicotinic acetylcholine receptor in healthy young participants Values are presented as mean (95% confidence interval). Group comparisons between male and female participants were performed using Welch’s t-test. Effect size is reported as Hedges’ g. A p value of <0.05 was considered statistically significant Alpha7 nAChR: serum levels of alpha7 nicotinic acetylcholine receptor; CI: confidence interval

Parameter	Participants (n = 33)	Male (n = 18)	Female (n = 15)	Mean difference (95% CI)	Test statistic	df	p value	Effect size
Alpha7 nAChR (ng/mL)	137.04 (128.78-145.31)	138.24 (126.81-149.67)	135.61 (122.08-149.13)	-2.63 (-19.63 to 14.36)	-0.32	29	0.754	g = -0.11

The comparison of HRV and autonomic reactivity parameters between healthy young men (n = 18) and women (n = 15) is summarized in Table 3. No statistically significant sex differences were observed for most HRV measures, with the exception of systolic BP (SBP) and diastolic BP (DBP), which were significantly higher in men (median SBP: 118.5 vs. 105.0 mmHg; median difference = -14.00 mmHg, 95% CI: -19.00 to -8.00, p < 0.001; median DBP: 72.5 vs. 67.0 mmHg, median difference = -7.00 mmHg, 95% CI: -13.00 to -3.00, p = 0.004). Although nonsignificant, women exhibited higher values in parasympathetic-associated indices such as RMSSD (median difference = +10.40 ms, 95% CI: -3.90 to 25.30, p = 0.193), NN50 (+41.50 counts, 95% CI: -6.00 to 84.00, p = 0.096), HF power (+477.50 ms², 95% CI: -190.00 to 1244.00, p = 0.178), and SD1 (+7.35 ms, 95% CI: -2.80 to 17.80, p = 0.206). Conversely, men showed slightly higher sympathetic indicators such as LF/HF ratio and LF normalized units, though these differences did not reach statistical significance (LF/HF median difference = -0.32, 95% CI: -0.68 to 0.02, p = 0.062; LF nu = -11.45, 95% CI: -24.19 to 0.59, p = 0.062). Other HRV parameters and autonomic reactivity tests (including ΔDBP during IHG and E:I ratio) did not differ significantly between sexes. Effect sizes ranged from small to moderate across comparisons, consistent with subtle sex-based physiological differences.

**Table 3:** Comparison of heart rate variability and autonomic reactivity test results between healthy young male and female participants All values are presented as median (IQR). Median differences (95% CI) are calculated using the Hodges-Lehmann estimator. Comparisons between the groups were performed using the Mann-Whitney U test. Effect sizes are reported as rank-biserial correlations ^*^p values of <0.05 were considered statistically significant Mean RR: average RR interval between the heart beats; SDNN: standard deviation of NN intervals; RMSSD: square root of the mean squared differences of successive NN intervals; NN50: number of pairs of adjacent NN intervals differing by more than 50 ms; LF: low-frequency power; HF: high-frequency power; TP: total power; LF nu: low-frequency power in normalized units, (LF / (TP - VLF)) × 100; HF nu: high-frequency power in normalized units, (HF / (TP − VLF)) × 100; SampEn: sample entropy; ApEn: approximate entropy; SD1: standard deviation of RR intervals perpendicular to the line of identity in the Poincaré plot; SD2: standard deviation of RR intervals along the line-of-identity in the Poincaré plot; SD2/SD1: ratio of SD2 to SD1; SBP: systolic blood pressure; DBP: diastolic blood pressure; HR: heart rate; RPP: rate pressure product, calculated as (SBP × HR) / 100; an index of myocardial oxygen demand; ΔDBP in IHG: change in diastolic blood pressure during the isometric handgrip (IHG); E:I ratio: ratio of mean RR intervals during expiration to inspiration in the deep breathing test; IQR: interquartile range; CI: confidence interval

Parameter	Participants (n = 33)	Male (n = 18)	Female (n = 15)	Median difference (95% CI)	Mann-Whitney U	p value	Rank-Biserial correlation
Mean RR (ms)	819.00 (767.00-874.00)	832.00 (788.00-871.50)	804.00 (764.50-873.50)	-24.00 (-83.00 to 40.00)	113.00	0.442	0.16
SDNN (ms)	58.90 (47.00-73.00)	58.15 (46.85-67.27)	61.10 (52.20-78.28)	7.30 (-7.00 to 21.30)	162.00	0.343	-0.20
RMSSD (ms)	52.20 (35.50-67.00)	44.50 (34.53-57.33)	53.80 (44.70-76.40)	10.40 (-3.90 to 25.30)	171.50	0.193	-0.27
NN50 (count)	105.00 (54.00-143.00)	78.50 (51.75-123.50)	110.00 (93.50-170.00)	41.50 (-6.00 to 84.00)	181.50	0.096	-0.34
LF (ms^2^)	654.00 (427.00-1,148.00)	671.00 (465.25-1,147.50)	654.00 (390.00-1,354.00)	-56.00 (-340.00 to 445.00)	130.00	0.873	0.04
HF (ms^2^)	1,206.00 (541.00-1,683.00)	1,012.50 (491.50-1,569.50)	1,217.00 (880.00-2,528.00)	477.50 (-190.00 to 1,244.00)	173.00	0.178	-0.28
TP (ms^2^)	3,154.00 (2,183.00-4,557.00)	3,046.50 (1,990.25-4,271.75)	3,154.00 (2,343.00-5,237.00)	539.00 (-863.00 to 2,085.00)	156.00	0.464	-0.16
LF/HF	0.65 (0.34-1.10)	0.87 (0.63-1.26)	0.58 (0.28-0.81)	-0.32 (-0.68 to 0.02)	83.00	0.062	0.39
LF nu	39.49 (25.26-52.26)	46.47 (38.47-55.80)	36.79 (21.49-44.61)	-11.45 (-24.19 to 0.59)	83.00	0.062	0.39
HF nu	60.48 (47.67-74.73)	53.52 (44.19-61.28)	63.02 (55.36-78.22)	11.36 (-0.42 to 24.43)	186.00	0.067	-0.38
SampEn	1.70 (1.60-1.79)	1.73 (1.65-1.79)	1.64 (1.57-1.78)	-0.09 (-0.20 to 0.06)	100.50	0.219	0.26
ApEn	1.17 (1.14-1.21)	1.19 (1.14-1.22)	1.16 (1.13-1.20)	-0.01 (-0.06 to 0.04)	120.50	0.613	0.11
SD1 (ms)	37.00 (25.10-47.50)	31.50 (24.42-40.57)	38.10 (31.70-54.10)	7.35 (-2.80 to 17.80)	170.50	0.206	-0.26
SD2 (ms)	77.00 (61.00-91.00)	75.50 (60.22-87.28)	79.60 (63.15-99.45)	6.95 (-10.20 to 24.60)	160.00	0.376	-0.19
SD2/SD1	2.20 (1.69-2.43)	2.28 (2.09-2.53)	1.74 (1.63-2.37)	-0.38 (-0.71 to 0.11)	93.00	0.135	0.31
Resting parameters							
SBP (mmHg)	113.00 (105.00-119.00)	118.50 (114.50-125.00)	105.00 (102.00-111.00)	-14.00 (-19.00 to -8.00)	22.50	<0.001^*^	0.83
DBP (mmHg)	70.00 (67.00-76.00)	72.50 (70.25-80.75)	67.00 (63.00-69.50)	-7.00 (-13.00 to -3.00)	56.00	0.004^*^	0.59
HR (bpm)	73.00 (67.00-78.00)	72.00 (67.25-77.00)	73.00 (67.00-82.50)	2.00 (-5.00 to 10.00)	149.50	0.612	-0.11
RPP (%)	83.78 (72.75-92.96)	85.55 (74.84-96.36)	75.48 (68.78-88.27)	-7.00 (-17.94 to 1.97)	92.00	0.126	0.32
Reactivity tests							
ΔDBP in IHG (mmHg)	20.00 (13.00-26.00)	20.00 (9.75-25.25)	19.00 (15.00-24.50)	2.00 (-6.00 to 11.00)	149.00	0.625	-0.10
E:I ratio	1.45 (1.36-1.57)	1.44 (1.40-1.56)	1.47 (1.33-1.58)	0.00 (-0.10 to 0.13)	135.00	1.000	0.00

As shown in Table 4, alpha7 nAChR levels were not significantly correlated with age (ρ = -0.02, 95% CI: -0.41 to 0.38, p = 0.898) or WHO-5 Well-Being Index scores (ρ = 0.24, 95% CI: -0.10 to 0.55, p = 0.181) in the overall sample. A negative trend was observed between alpha7 nAChR and BMI (ρ = -0.35, 95% CI: -0.61 to 0.01, p = 0.053), though this did not reach statistical significance. In sex-stratified analyses, this trend appeared more pronounced in men (ρ = -0.45, 95% CI: -0.73 to 0.00, p = 0.063), while no significant associations were found in women. These findings suggest a possible sex-specific relationship between peripheral cholinergic markers and adiposity, warranting further investigation in larger cohorts.

**Table 4:** Correlation of alpha7 nicotinic acetylcholine receptor levels with age, BMI, and WHO Well-Being Index scores in healthy young participants Spearman’s rank correlation coefficients (ρ) and corresponding 95% CIs were obtained via 1,000 bootstrap samples. Analyses were conducted for the full sample and separately for male and female participants. Z-statistics and p values were calculated using Fisher’s Z transformation. A p value of <0.05 was considered statistically significant. The WHO-5 score has been referred from [12] BMI: body mass index; WHO-5 score: World Health Organization Well-Being Index (five items) score; CIs: confidence intervals

Parameter	Spearman's ρ (all) (95% CI)	Z-statistic	p value	Spearman's ρ (male) (95% CI)	Z-statistic	p value	Spearman's ρ (female) (95% CI)	Z-statistic	p value
Age	-0.02 (-0.41 to 0.38)	-0.11	0.898	0.31 (-0.27 to 0.74)	1.25	0.208	-0.3 (-0.82 to 0.32)	-1.08	0.272
BMI (kg/m^2^)	-0.35 (-0.61 to 0.01)	-2.00	0.053	-0.45 (-0.73 to 0)	-1.86	0.063	-0.09 (-0.6 to 0.55)	-0.32	0.746
WHO-5 score	0.24 (-0.1 to 0.55)	1.34	0.181	0.39 (-0.08 to 0.7)	1.58	0.112	-0.03 (-0.61 to 0.59)	-0.09	0.924

Correlation analyses between serum alpha7 nAChR levels and HRV parameters are summarized in Table 5. In the overall sample and within sex-specific subgroups, no statistically significant correlations were found between alpha7 nAChR levels and any HRV metric (all p values >0.05). Spearman's partial correlations were used, adjusting for age and BMI to control for potential confounding factors. To increase the estimate reliability, 95% CIs were derived from 1,000 bootstrap samples. Despite this robust nonparametric approach, which is well-suited for small sample sizes and nonnormal data distributions, none of the observed associations approached statistical significance. These findings indicate that serum alpha7 nAChR levels do not exhibit meaningful relationships with resting autonomic function, as assessed by HRV indices, in healthy young adults. The consistency of correlation direction and magnitude across both men and women further reinforces these null results.

**Table 5:** Correlation between alpha7 nicotinic acetylcholine receptor levels and heart rate variability parameters in young healthy participants Correlation analyses between serum alpha 7 nicotinic acetylcholine receptor levels and HRV parameters were performed using Spearman’s partial correlation, controlling for age and BMI in a stepwise manner to minimize collinearity bias. Results are reported as Spearman’s ρ with 95% confidence intervals (based on 1,000 bootstrap samples). Z-statistics and p values were calculated using Fisher’s Z transformation. A p value of <0.05 was considered statistically significant Mean RR: average RR interval between the heart beats; SDNN: standard deviation of NN intervals; RMSSD: square root of the mean squared differences of successive NN intervals; NN50: number of pairs of adjacent NN intervals differing by more than 50 ms; LF: low-frequency power; HF: high-frequency power; TP: total power; LF nu: low-frequency power in normalized units, (LF / (TP - VLF)) × 100; HF nu: high-frequency power in normalized units, (HF / (TP − VLF)) × 100; SampEn: sample entropy; ApEn: approximate entropy; SD1: standard deviation of RR intervals perpendicular to the line of identity in the Poincaré plot; SD2: standard deviation of RR intervals along the line-of-identity in the Poincaré plot; SD2/SD1: ratio of SD2 to SD1; HRV: heart rate variability; BMI: body mass index

Parameter	Spearman's ρ (all) (95% CI)	Z-statistic	p value	Spearman's ρ (male) (95% CI)	Z-statistic	p value	Spearman's ρ (female) (95% CI)	Z-statistic	p value
Mean RR (ms)	0.128 (-0.25 to 0.473)	0.685	0.499	-0.005 (-0.516 to 0.508)	-0.021	0.984	0.117 (-0.49 to 0.648)	0.392	0.703
SDNN (ms)	0.002 (-0.365 to 0.368)	0.009	0.993	-0.164 (-0.624 to 0.38)	-0.623	0.543	0.032 (-0.552 to 0.595)	0.105	0.918
RMSSD (ms)	0.024 (-0.345 to 0.387)	0.129	0.898	-0.241 (-0.67 to 0.31)	-0.928	0.369	0.151 (-0.463 to 0.667)	0.508	0.621
NN50 (count)	0.175 (-0.204 to 0.509)	0.943	0.354	0.012 (-0.503 to 0.521)	0.046	0.964	0.192 (-0.429 to 0.69)	0.650	0.529
LF (ms^2^)	0.207 (-0.172 to 0.533)	1.121	0.272	0.104 (-0.431 to 0.585)	0.393	0.7	0.071 (-0.524 to 0.62)	0.237	0.817
HF (ms^2^)	0.15 (-0.229 to 0.49)	0.805	0.427	-0.01 (-0.52 to 0.505)	-0.039	0.97	0.116 (-0.49 to 0.647)	0.389	0.705
TP (ms^2^)	0.055 (-0.318 to 0.414)	0.294	0.771	-0.054 (-0.551 to 0.471)	-0.203	0.842	-0.133 (-0.657 to 0.477)	-0.445	0.665
LF/HF	0.047 (-0.325 to 0.407)	0.250	0.805	0.238 (-0.312 to 0.669)	0.918	0.374	-0.086 (-0.629 to 0.513)	-0.287	0.78
LF nu	0.047 (-0.325 to 0.407)	0.250	0.805	0.238 (-0.312 to 0.669)	0.918	0.374	-0.086 (-0.629 to 0.513)	-0.287	0.78
HF nu	-0.046 (-0.405 to 0.326)	-0.242	0.81	-0.225 (-0.661 to 0.324)	-0.865	0.402	0.086 (-0.513 to 0.629)	0.287	0.78
SampEn	0.168 (-0.211 to 0.504)	0.904	0.374	0.024 (-0.494 to 0.53)	0.091	0.929	0.312 (-0.319 to 0.751)	1.090	0.299
ApEn	0.094 (-0.282 to 0.445)	0.499	0.622	0.16 (-0.384 to 0.621)	0.605	0.555	0.139 (-0.473 to 0.66)	0.465	0.651
SD1 (ms)	0.033 (-0.337 to 0.395)	0.176	0.862	-0.241 (-0.67 to 0.31)	-0.928	0.369	0.173 (-0.445 to 0.679)	0.582	0.572
SD2 (ms)	-0.001 (-0.367 to 0.366)	-0.006	0.995	-0.157 (-0.619 to 0.386)	-0.595	0.562	-0.007 (-0.579 to 0.569)	-0.024	0.981
SD2/SD1	-0.067 (-0.423 to 0.307)	-0.357	0.724	0.298 (-0.253 to 0.703)	1.168	0.262	-0.365 (-0.776 to 0.265)	-1.298	0.221

Correlation analyses between serum alpha7 nAChR levels and autonomic reactivity test parameters are summarized in Table 6. A statistically significant positive association was observed between alpha7 nAChR levels and the E:I ratio, an established index of parasympathetic reactivity during deep breathing. This association was present in the combined sample (ρ = 0.384, 95% CI: 0.021-0.658, p = 0.036) and was more pronounced in men (ρ = 0.573, 95% CI: 0.086-0.839, p = 0.020). No significant correlations were identified between alpha7 nAChR levels and resting SBP, DBP, heart rate (HR), rate pressure product (RPP), or the change in DBP during IHG (ΔDBP in IHG) in either sex or the overall cohort (p > 0.05 for all). All analyses controlled for age and BMI to minimize confounding, and 95% CIs were derived from 1,000 bootstrap resamples to enhance the robustness of the estimates. Notably, the E:I ratio remained the only autonomic parameter significantly associated with alpha7 nAChR levels, suggesting a specific link between receptor expression and cardiovagal reactivity, particularly in men.

**Table 6:** Correlation between alpha7 nicotinic acetylcholine receptor levels and autonomic reactivity test results in healthy young participants Correlation analyses between serum alpha7 nicotinic acetylcholine receptor levels and both resting and autonomic reactivity test parameters were conducted using Spearman’s partial correlation, controlling for age and BMI. Results are reported as Spearman’s ρ with 95% confidence intervals (based on 1,000 bootstrap samples). Z-statistics and corresponding p values were computed using Fisher’s Z transformation ^*^A p value of <0.05 was considered statistically significant SBP: systolic blood pressure; DBP: diastolic blood pressure; HR: heart rate; RPP: rate pressure product, calculated as (SBP × HR) / 100; an index of myocardial oxygen demand; ΔDBP in IHG: change in diastolic blood pressure during the isometric handgrip (IHG); E:I ratio: ratio of mean RR intervals during expiration to inspiration in the deep breathing test

Parameter	Spearman's ρ (all) (95% CI)	Z-statistic	p value	Spearman's ρ (male) (95% CI)	Z-statistic	p value	Spearman's ρ (female) (95% CI)	Z-statistic	p value
Resting parameters									
SBP (mmHg)	0.217 (-0.163 to 0.54)	1.175	0.25	0.344 (-0.204 to 0.728)	1.372	0.192	0.215 (-0.409 to 0.702)	0.730	0.481
DBP (mmHg)	0.239 (-0.14 to 0.557)	1.302	0.204	0.454 (-0.076 to 0.784)	1.908	0.077	-0.048 (-0.605 to 0.541)	-0.161	0.875
HR (bpm)	-0.016 (-0.381 to 0.352)	-0.086	0.932	0.029 (-0.491 to 0.533)	0.109	0.915	0.049 (-0.54 to 0.606)	0.162	0.874
RPP (%)	0.145 (-0.234 to 0.486)	0.778	0.443	0.251 (-0.3 to 0.676)	0.971	0.348	0.194 (-0.428 to 0.691)	0.656	0.525
Response in autonomic reactivity tests									
ΔDBP in IHG (mmHg)	-0.115 (-0.462 to 0.262)	-0.614	0.544	-0.389 (-0.752 to 0.154)	-1.581	0.136	0.176 (-0.442 to 0.681)	0.594	0.565
E:I ratio	0.384 (0.021 to 0.658)	2.202	0.036^*^	0.573 (0.086 to 0.839)	2.617	0.02^*^	0.213 (-0.412 to 0.701)	0.721	0.486

## Discussion

This study examined serum levels of alpha7 nAChR and their association with cardiovascular autonomic function in healthy young adults. A central focus was placed on potential sex-related differences, aiming to advance understanding of the receptor’s role in autonomic regulation, particularly in relation to parasympathetic and sympathetic activity at rest and reactivity tests. To reduce the influence of known confounding factors such as age-related autonomic decline or metabolic dysregulation, the sample was carefully selected to include only young, nonobese, and otherwise healthy individuals. By excluding participants with extreme age ranges and elevated BMI, we ensured a more physiologically homogeneous cohort, thus enhancing the internal validity of our findings.

Demographic analysis and participant homogeneity

As shown in Table 1, the demographic characteristics were well balanced between men and women. No significant sex differences were observed in age, BMI, or WHO-5 Well-Being Index scores. The average participant age was 22.76 ± 3.38 years, and BMI values were comparable across sexes. This uniformity in key demographic and psychological health variables reinforces the reliability of between-group comparisons of serum alpha7 nAChR levels and autonomic measures. Importantly, all participants scored above 50 on the WHO-5 Well-Being Index, indicating a generally positive psychological state and a low likelihood of depressive symptoms or emotional distress. This adds another layer of sample consistency, as mood and mental health are known to modulate autonomic tone and potentially interact with cholinergic signaling pathways. Including only psychologically stable individuals thus strengthens the interpretability of associations involving autonomic and alpha7 nAChR-related parameters.

Serum alpha7 nicotinic acetylcholine receptor levels

Alpha7 nicotinic acetylcholine receptors (alpha7 nAChR) are integral to the cholinergic anti-inflammatory pathway and are expressed across diverse cell types, including those involved in vascular and immune functions [3-6]. Our study is among the first to report circulating serum alpha7 nAChR levels in healthy humans, providing novel baseline data for this biomarker. In this young, healthy cohort, serum alpha7 nAChR concentrations averaged 137.04 ng/mL (95% CI: 128.78-145.31), with moderate interindividual variability reflected by coefficients of variation around 17%. Although men exhibited slightly higher mean levels (138.24 ng/mL; 95% CI: 126.81-149.67) than women (135.61 ng/mL; 95% CI: 122.08-149.13), the difference was not statistically significant (mean difference = -2.63 ng/mL, 95% CI: -19.63 to 14.36; p = 0.754; Hedges’ g = -0.11). This suggests that, within a young and metabolically healthy population, serum alpha7 nAChR expression is relatively stable and does not substantially differ by sex. These findings provide an important reference point for future studies investigating alpha7 nAChR as a potential biomarker in cardiovascular and inflammatory conditions. Larger and more diverse samples, including wider age ranges and hormonal influences, are warranted to further clarify how these factors may modulate circulating alpha7 nAChR levels.

Resting HRV and autonomic function tests

HRV offers valuable insights into autonomic regulation by reflecting the dynamic balance between sympathetic and parasympathetic activity. In this study, we evaluated multiple HRV parameters, including mean RR intervals, SDNN, RMSSD, NN50, LF power, HF power, total power, and nonlinear indices like SD1 and SD2, to comprehensively assess cardiac autonomic modulation. We did not apply formal corrections for multiple comparisons to avoid an undue increase in Type II error (false negatives). However, we acknowledge the increased risk of Type I errors (false positives) due to the number of HRV metrics analyzed. Our results did not reveal statistically significant sex differences in these HRV measures (Table 3). That said, several parameters associated with parasympathetic activity, namely RMSSD, HF power, and SD1, tended to be higher in women than in men. Although these differences did not reach statistical significance, their direction aligns with previous studies reporting greater vagal tone in women [14], which is likely influenced by estrogen’s modulatory effects on ANS activity.

In contrast, SBP and DBP were significantly higher in men than women, with median differences of -14.00 and -7.00 mmHg, respectively (p < 0.001 and p = 0.004; Table 3). An ANCOVA controlling for BMI confirmed that sex differences in both SBP and DBP persisted: for SBP, gender, F(1, 30) = 40.76, p < 0.001, and BMI, F(1, 30) = 7.81, p = 0.009, accounted for approximately 58% and 21% of the variance, respectively. For DBP, gender remained a significant predictor, F(1, 30) = 12.88, p = 0.001, explaining around 30% of the variance, while BMI showed a nonsignificant trend, F(1, 30) = 3.69, p = 0.064, accounting for about 11%. These results indicate that sex differences in both SBP and DBP persist after adjusting for BMI, with a stronger influence observed for systolic pressure. This finding is consistent with previous literature [15] and may relate to sex-specific effects of gonadal hormones, body composition, and lifestyle factors influencing cardiovascular function [16]. However, since we did not collect detailed data on physical activity or diet, we could not adjust BP analyses for these variables. No significant sex differences were observed in resting heart rate or RPP. Although some studies have reported higher resting heart rates in women, possibly as a compensatory mechanism to maintain cardiac output despite lower BP [14], our data did not reflect this pattern. This may be due to our relatively small sample size and the narrow BMI and age ranges studied. Notably, despite higher SBP in men, the modest and nonsignificant increase in RPP is likely explained by similar heart rates between the groups.

Autonomic reactivity was assessed via the IHG test and the deep breathing test. The change in DBP during IHG was similar between men and women, suggesting comparable sympathetic reactivity. This aligns with findings by Kodzo et al., who also reported no significant sex differences in IHG-induced DBP responses [17]. While some studies have reported greater DBP responses in men [18,19], these often involved participants with higher BMI or broader age ranges (e.g., 20-50 years), complicating direct comparisons. The deep breathing test, assessing parasympathetic reactivity, also showed no significant sex difference in the E:I ratio. Although some prior reports suggest women have higher E:I ratios [19], our results are consistent with normative data from the Indian population, which show no marked sex differences [20].

Taken together, these results suggest that while resting BP clearly differs between sexes, autonomic reactivity, covering both sympathetic and parasympathetic responses, remains similar in healthy young men and women. This implies a stable resting autonomic regulation across sexes within this age group in our study population.

Correlation between alpha7 nAChR levels and demographic variables

We investigated the correlations between serum alpha7 nAChR levels and some demographic and health indicators, including age, BMI, and World Health Organization Well-Being Index (five items) scores (Table 4). In the overall sample, no significant correlation was observed between alpha7 nAChR levels and age. However, stratified analyses revealed a positive correlation trend in men and a negative trend in women. Although these gender-specific patterns did not reach statistical significance, they may suggest hormonal modulation of receptor expression and regulation.

To further explore these observations, a robust linear regression was performed to assess the effects of age, gender, and their interaction on alpha7 nAChR levels. The age × gender interaction term showed a positive coefficient (β = 4.69, SE = 3.53, t = 1.33), indicating a potential gender-specific age effect, with alpha7 nAChR levels possibly increasing with age in men relative to women. However, this did not achieve statistical significance, likely due to the limited age range (18-35 years) and modest sample size. Future studies with a broader age distribution and larger cohorts are needed to clarify these potential sex differences in age-related receptor changes.

BMI demonstrated a weak negative correlation with alpha7 nAChR levels in the full sample (ρ = -0.35, 95% CI: -0.61 to 0.01, p = 0.053), approaching but not reaching statistical significance. This negative association was more pronounced in men (ρ = -0.45, 95% CI: -0.73 to 0.00, p = 0.063), compared to women (ρ = -0.09, 95% CI: -0.6 to 0.55, p = 0.746), though still nonsignificant. Given the higher prevalence of proinflammatory states in men [21] and the role of alpha7 nAChR in modulating inflammation [22,23], particularly within adipocytes, male gonadal hormones might influence both inflammatory status and BMI. This could underlie the observed inverse relationship between alpha7 nAChR levels and BMI in men [24]. Moreover, previous research has documented downregulation of alpha7 nAChR expression in adipocytes with obesity and its restoration following weight loss [24], which may further explain this correlation. These findings point to a potential sex-specific link between adiposity and alpha7 nAChR regulation, meriting deeper investigation. It should be noted, however, that BMI is a crude measure of adiposity; more detailed assessments of body composition would provide greater insight into the receptor’s relationship with adipose tissue.

Finally, WHO-5 Well-Being Index scores, reflecting subjective quality of life, showed no significant correlation with alpha7 nAChR levels in either the overall sample or when analyzed by sex. This suggests that in young, healthy individuals, serum alpha7 nAChR levels are not directly associated with psychological or subjective well-being.

Correlation between alpha7 nAChR levels and HRV parameters

We examined the correlations between serum alpha7 nicotinic acetylcholine receptor (alpha7 nAChR) levels and HRV parameters, adjusting for age and BMI via partial correlation analyses (Table 5). Although most correlations at rest did not reach statistical significance, a consistent sex-specific pattern emerged, suggesting that these relationships are independent of confounding by age and BMI.

In men, parasympathetic markers such as RMSSD, high-frequency power in normalized units, and SD1 tended to correlate negatively with alpha7 nAChR levels, whereas predominantly sympathetic indices, such as LF nu, LF/HF, and DBP, showed positive associations. Conversely, women exhibited the opposite pattern: alpha7 nAChR levels were positively associated with parasympathetic indices and negatively with sympathetic markers. Estrogen is known to enhance vagal tone and modulate inflammatory processes, which could theoretically influence cholinergic-autonomic interactions. However, all female participants were assessed during the early proliferative phase of the menstrual cycle, when estrogen levels are relatively low, and estrogen was not directly measured in this study. Therefore, attributing these sex-specific differences solely to hormonal influences remains speculative. Nevertheless, prior research supports estrogen’s role in modulating vagal activity [14], making it a plausible contributing factor.

Post hoc power analysis indicated that while the overall sample had sufficient power to detect moderate correlations, power was limited within sex-specific subgroups, which may partially explain the lack of statistical significance for some HRV parameters. This limitation should be taken into account when interpreting subgroup findings. The consistency of bootstrap-derived CIs across sexes, despite nonsignificant p-values, supports the internal reliability of the observed trends. This methodological approach strengthens confidence in the findings, particularly given the small sample size and exploratory nature of the analyses.

While our sample size may have constrained the detection of statistically significant associations, the observed sex-specific trends merit further investigation. The role of alpha7 nAChR in autonomic regulation may be more pronounced under pathological conditions or in populations with altered metabolic or cardiovascular health, highlighting avenues for future research.

Correlation between alpha7 nAChR levels and autonomic reactivity

**Figure 1:**
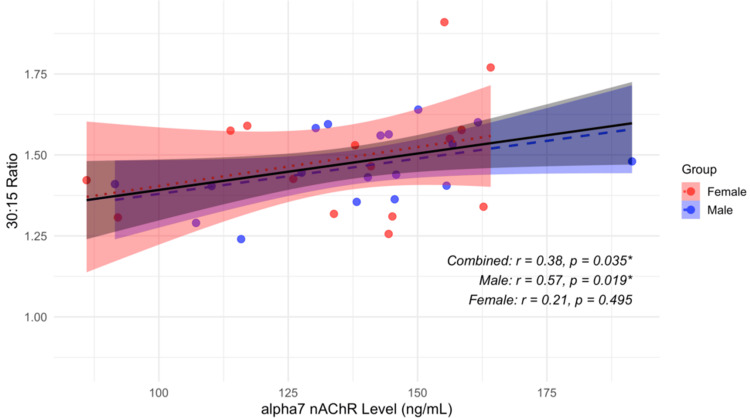
Scatterplot of serum alpha7 nAChR levels and 30:15 ratio by sex Scatterplot illustrating partial correlations between serum alpha7 nAChR levels and the 30:15 ratio, a marker of parasympathetic reactivity, in healthy young adults. Data are shown separately for men (blue), women (red), and the combined cohort (black). Linear regression lines with 95% confidence bands are overlaid for each group. Values are adjusted for age and BMI. The inset displays correlation coefficients (r) and p values: ^*^p < 0.05 (uncorrected). The association in men remained significant after FDR correction (adjusted p = 0.041) alpha7 nAChR: alpha7 nicotinic acetylcholine receptor

Our findings indicate no significant relationship between serum alpha7 nAChR levels and resting cardiovascular parameters, including SBP, DBP, HR, and RPP, after 15 minutes of rest (Table 6). Similarly, no association was observed with the ΔDBP response to IHG, a sympathetic reactivity measure, across genders. These results suggest that alpha7 nAChR expression does not influence resting autonomic vascular or cardiac function in healthy young adults. However, a significant sex-specific association emerged during the deep breathing test (Figure 1).

The E:I ratio, a marker of parasympathetic (vagal) reactivity, correlated positively with alpha7 nAChR levels in the overall sample (ρ = 0.384, 95% CI: 0.021-0.658, p = 0.036) and more strongly in men (ρ = 0.573, 95% CI: 0.086-0.839, p = 0.020), but not significantly in women (ρ = 0.213, 95% CI: -0.412 to 0.701, p = 0.486). This relationship persisted in men even after adjusting for multiple comparisons using false discovery rate (FDR) correction (adjusted p = 0.041). These data suggest that higher alpha7 nAChR expression may be linked to enhanced parasympathetic cardiac modulation during deep breathing in men.

Alpha7 nAChR is a critical component of the cholinergic pathway, mediating vagus nerve-driven signaling and expressed within central autonomic and respiratory centers that regulate HRV during respiratory cycles [24,25]. Therefore, elevated alpha7 nAChR levels may reflect more robust vagal cholinergic signaling capacity, which is more apparent in men in this cohort. Gonadal hormones and cytokines may modulate this interaction [26-29]. However, since all female participants were tested during the early proliferative phase, characterized by comparatively lower estrogen levels, and hormone measurements were not conducted, the exact role of hormonal influences remains uncertain. Notably, despite sex differences in receptor levels, the direction of correlation between alpha7 nAChR and parasympathetic reactivity was consistent across sexes, differing from patterns observed in resting parasympathetic tone.

Importantly, no association was found with sympathetic reactivity as measured by ΔDBP during IHG, highlighting the specificity of alpha7 nAChR’s involvement in parasympathetic rather than generalized autonomic modulation. This specificity further underscores the potential role of alpha7 nAChR in cardiac vagal regulation.

Overall, these findings support a gender-specific modulation of vagal tone via alpha7 nAChR signaling and warrant further investigation to elucidate underlying mechanisms and explore implications in clinical populations with autonomic dysregulation.

Limitations of the study

This study has several limitations that should be considered. Although the sample size was modest, all 33 participants initially calculated for the parent study were included in the analysis, and the study was adequately powered for exploratory investigation. Even so, the findings should be interpreted with caution, and replication in larger cohorts is needed to confirm these preliminary results. While efforts were made to reduce hormonal variability by including only female participants in the early proliferative phase of the menstrual cycle and excluding those on hormonal contraceptives, we did not collect direct measures of hormone levels. This limits our ability to fully assess the role of hormonal influences in the observed gender-specific associations. In addition, we did not measure cytokines, protein expression, or lifestyle factors such as diet, physical activity, smoking, or alcohol use, which may also impact autonomic and cholinergic pathways.

Although short-term (five-minute) HRV recordings are well-established for assessing parasympathetic and sympathetic modulation, longer recordings might have provided additional insight into slower regulatory processes. Furthermore, given the number of correlations examined, there is a risk of false-positive findings. While we applied FDR correction for key autonomic reactivity outcomes, we did not adjust for all comparisons, in keeping with the exploratory nature of the study. The cross-sectional design also limits our ability to infer causality between alpha7 nAChR levels and autonomic function. Finally, the relatively homogeneous sample, composed of healthy young adults with normal BMI, limits the generalizability of our findings to other populations, including older individuals or those with cardiometabolic conditions. Future studies should aim to address these limitations by including larger, more diverse samples, longitudinal designs, and relevant biochemical markers to better elucidate underlying mechanisms.

## Conclusions

Serum levels of alpha7 nAChR did not differ significantly between male and female participants. A trend toward a negative correlation with BMI was observed, particularly in male participants, though this finding should be interpreted cautiously given the limited sample size and borderline statistical significance. While no consistent associations were found between alpha7 nAChR levels and resting HRV parameters, a positive correlation with the E:I ratio during deep-breathing was observed in male participants, and this association remained significant after correction for multiple testing.

These preliminary, sex-specific patterns suggest possible links between alpha7 nAChR signaling and autonomic function that warrant further investigation. Given the exploratory nature of this study, the results should be viewed as hypothesis-generating rather than confirmatory. Future studies incorporating larger, more diverse cohorts and longitudinal designs are needed to better understand the temporal dynamics and potential mechanistic pathways involved. While these early findings may hold translational relevance, especially in the context of autonomic and inflammatory regulation, they remain preliminary and are not yet clinically actionable.
